# Distraction of olfactory bulb-medial prefrontal cortex circuit may induce anxiety-like behavior in allergic rhinitis

**DOI:** 10.1371/journal.pone.0221978

**Published:** 2019-09-11

**Authors:** Morteza Salimi, Sepideh Ghazvineh, Meysam Zare, Tannaz Parsazadegan, Kolsum Dehdar, Milad Nazari, Javad Mirnajafi-Zadeh, Hamidreza Jamaati, Mohammad Reza Raoufy

**Affiliations:** 1 Department of Physiology, Faculty of Medical Sciences, Tarbiat Modares University, Tehran, Iran; 2 Faculty of Electrical Engineering, Sharif University of Technology, Tehran, Iran; 3 Institute for Brain Sciences and Cognition, Faculty of Medical Sciences, Tarbiat Modares University, Tehran, Iran; 4 Chronic Respiratory Diseases Research Center, National Research Institute of Tuberculosis and Lung Diseases, Shahid Beheshti University of Medical Sciences, Tehran, Iran; Technion Israel Institute of Technology, ISRAEL

## Abstract

Allergic rhinitis is a chronic inflammatory disease of the upper respiratory tract, which is associated with high incidence of anxiety symptom. There is evidence that medial prefrontal cortex modulates anxiety-related behaviors and receives projections from olfactory bulb. Since olfactory dysfunction has been reported in allergic rhinitis, we aimed to evaluate anxiety-like behavior and oscillations of olfactory bulb-medial prefrontal cortex circuit in an animal model of allergic rhinitis. The number of open arm entries in elevated zero maze was significantly reduced in sensitized rats exposed to intranasal ovalbumin compared to the control group, which was indicating the enhancement of anxiety-like behavior in allergic rhinitis animals. Analysis of local field potentials in olfactory bulb and medial prefrontal cortex during immobility and exploration state showed that anxiety-like behavior induced by allergic rhinitis was in association with increased activity of medial prefrontal cortex and enhancement of olfactory bulb-medial prefrontal cortex coupling in delta and theta bands. Moreover, in allergic rhinitis animals, theta strongly coordinates local gamma activity in olfactory bulb and medial prefrontal cortex, which means to have a strong local theta/gamma coupling. We suggested that disruption of olfactory bulb-medial prefrontal cortex circuit due to allergic reactions might have a governing role for inducing anxiety-like behavior in the allergic rhinitis experimental model.

## Introduction

Allergic rhinitis (AR) is a chronic inflammatory disorder of the nasal mucosa, which is characterized by the presence of one or some symptoms, including itchiness, sneezing, rhinorrhea, nasal congestion, and daily alterations in sense of olfaction [1–4]. AR is a highly prevalent chronic disease that affects 400 million people worldwide; and epidemiological studies have reported that 20–30% of adults and 40% of children suffer from AR [5–7]. In addition to classical manifestations, patients with AR report decline of cognitive behavior, such as anxiety, reduced ability to concentrate, learning disability, and memory impairment [8–11]. These behavioral complications adversely affect the quality of life and social productivity [3]. Anxiety is the most common psychological problems related to allergic disorders and is associated with poor disease outcomes [12–15]. It has been proposed that antigen exposure raised the level of inflammatory mediators such as interleukin-1β, which stimulated the hypothalamic-pituitary-adrenal axis and cortisol activation [16]. These reactions are known to disrupt serotonin release and led to mood changes, and particularly induced the anxiety [17]. Furthermore, production of T helper cytokines, including TH2 and corticotropin-releasing factor in the medial prefrontal cortex (mPFC) and olfactory bulbs (OB) were associated with a heightened level of anxiety-like behaviors in AR rats [17]. AR model in rodents was associated with impairment of olfactory function, increased size and number of olfactory glands, and infiltration of inflammatory cells in the olfactory mucosa [18,19]. These alterations reduced local field potential (LFP) power of OB in the mouse model of AR [19]. Moreover, exposure to intranasal antigens in sensitized animals activated limbic brain regions, represented by neurons stained for the c-Fos protein [20,21]. In a human study, increased brain activity determined by magnetic resonance in the prefrontal cortex has been reported during the late phase of asthma episode [21].

Retrograde and anterograde tracing experiments confirmed anatomical and functional connections between OB and PFC in representing states of emotional reactivity [22]. A human study reported that patients with generalized anxiety disorder exhibited hyper-activation of the prefrontal cortex [23]. Likewise, electrophysiological recordings in animals revealed hyperactivity of mPFC in association with anxiety-related behaviors [24,25]. The prefrontal cortex has functional and structural connectivity to the olfactory pathway [22]. Nasal breathing via the olfactory bulb modulated neural oscillations in widespread subcortical and neocortical areas, including prefrontal cortex which were known as respiration entrained brain oscillations [26]. The olfactory bulb has a close link to limbic brain regions; and chronic removal of olfactory profoundly affects emotional behaviors such as anxiety [27,28]. A recent investigation in rodents suggested that prefrontal cortex oscillations during fear conditioning were correlated with respiratory/olfactory rhythms (~ 4 Hz) through the nasal cavity in induced freezing behavior [22].

Taken together, OB-mPFC functional connectivity seems to have a key role in anxiety behaviors, while previous studies in AR reported alteration in each of these regions separately by focusing on cellular and molecular methods. Therefore, we hypothesized that AR-induced anxiety-like behavior was associated with disruption of OB-mPFC functional connectivity. To investigate this hypothesis, we evaluated oscillation alteration in OB-mPFC circuit as well as anxiety like-behavior in a rat model of AR.

## Materials and methods

### Animals

Sixteen adult (2–4 months old) pathogen-free male Wistar rats weighing 80–100 g were obtained from the Pasteur Institute (Tehran, Iran). Animals were kept at 21 ± 2°C and 12-h light on/light off cycle (7 a.m. -7 p.m.). All animal protocols were reviewed and approved by the “Ethics Committee of Faculty of Medical Sciences, Tarbiat Modares University”.

### Sensitization protocol and experimental groups

**Fig 1:**
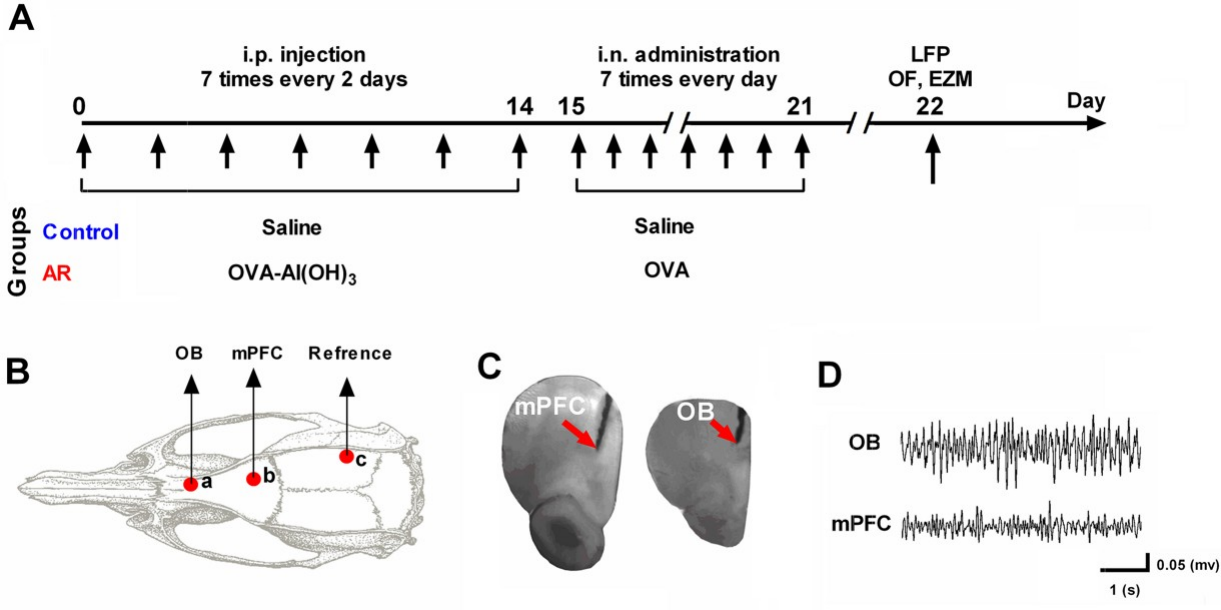
Schematic representation of the experimental design for AR rat model induced by OVA and electrode implantation. (A) Timeline of the study design. The rats received 7 intraperitoneal injections of saline or OVA-Al(OH)_3_ every 2 days from day 0 to day 14, and 7 intra-nasal injections every day from day 15 to day 21 with 10 μL of 10% OVA. (B) Schematic representation of electrode implantation sites on rat skull. (C) Histological confirmation of a recording site in OB and mPFC. (D) Representative traces of simultaneous LFP from OB and mPFC. AR: allergic rhinitis; OB: olfactory bulb; mPFC: medical prefrontal cortex; LFP: local field potential.

Rats were divided randomly into two groups including the control group and AR group (each group contained 8 animals). As described previously [29], in AR group, we sensitized the rats with ovalbumin (OVA) (0.3 mg i.p.) accompanied with Al(OH)_3_ (30 mg) in saline (1 ml i.p.) every 2 days for 14 days. Then, 10 μL of 10% OVA was administered during inspiration was administered every day to both nostrils from day 15 to day 21. In the control group, saline was administered to the rats, pending similar days (Fig 1A). To examine AR symptoms, rubbing were counted for 10 minutes on days 1, 14 and 21 by a blind observer to experimental procedures [29]

### Behavioral tests

Twenty-four hours after the last intranasal injection, open field and elevated zero maze (EZM) tests were conducted. We applied EZM test to estimate the level of anxiety in animals. The maze was 70 cm above the floor and made of an annular dark plexi (diameter of 120 cm, 10 cm wide circular corridor, and 30 cm high walls). The maze was divided into four equal quadrants, in which two opposite quadrants were open and the rest remained closed; animals were allowed to explore and move freely for 5 minutes. In the open field test, each rat was placed individually into a standard open field box (50 cm high, 75 × 75 cm) allowed to explore for 10 minutes. In the both tests, the movement of rats was recorded using a video camera and a graphical interface of MATLAB were applied for analyzing the percentage of spent time in the open quadrants and the number of open entries in the EZM, and the traveling distance for locomotor activity in open field test.

### Surgery

Animals were anesthetized with intraperitoneal injections of ketamine (100 mg/kg) and xylazine (10 mg/kg). Periodic injections of ketamine were carried out in order to have constant anesthesia throughout the surgery. Anesthesia induction was verified by the absence of withdrawal reflex followed by hind paw pinching. An intramuscular injection of 0.1 ml of dexamethasone (2.5 mg/ml) was applied to prevent swelling of the brain. Just before the surgery, 0.5 ml of lidocaine chlorhydrate 2% was subcutaneously applied in the scalp for local anesthesia. After anesthetic induction, animals were placed in a stereotaxic frame (Narishige, Japan) in a flat skull position. An incision was made on the scalp along the midline of the skull surface and then a stainless-steel recording electrodes (127 μm in diameter, A.M. system Inc., USA) were implanted unilaterally into the right hemisphere of two brain areas: OB (AP: 8.5 mm, ML:-1 mm, DV: -1.5 mm) and the prelimbic of mPFC (AP: +3.2mm; L: -0.6mm; DV: -3.6mm) [30]. We implanted a stainless-steel screw at the right side of parietal bone as a reference point (Fig 1B). The scalp skin was disinfected with antibiotics (tetracycline).

One week after surgery, rats were placed in an arena (50×50×50 cm) and LFPs were recorded. For this purpose, the socket, fixed on the animal’s head, was connected to a miniature buffer headstage with high-input impedance (BIODAC-A, TRITA WaveGram Co., Tehran, Iran), via cables to a main AC coupled amplifier (1000 amplification) and to the recording system (BIODAC-ESR18622, TRITA WaveGram Co., Tehran, Iran). Spontaneous LFPs simultaneously were recorded from OB and mPFC in immobility and exploration states of the awake rats. The signals were stored for offline processing using by custom-written MATLAB software (The Mathworks, Inc.). We applied a video tracker for recording the movement of animals to detect the different states.

### Histological verification

For conforming to the location of electrodes, the rats' brain were carefully removed and fixed with 4% paraformaldehyde for 48h. Then a coronal section of 200 mm thickness was prepared and visually compared to the matching slices in the rat's brain atlas of Paxinos and Watson [30] (Fig 1C). Animals with misplaced electrodes were excluded from the study.

### LFP analysis

We used Welch’s periodogram for computing power spectral density with MATLAB pwelch function. Coherence spectra between two regions were measured by calculating magnitude-squared coherence using mscohere function in MATLAB. Both power and coherence spectra computing were carried out in 30s segments of data by using 6 ms Hamming windows with 90% overlap. We calculated cross-correlation coefficient values for the delta (< 4 Hz) and theta (4–12 Hz) filtered signals by the mean of MATLAB xcorr function.

Phase-amplitude coupling was calculated using the framework which was previously described [31]. We analyzed the coupling between the phase of theta (4–12 Hz) bands and the amplitude of gamma band between 30 to 120 Hz. The phases of theta were binned into the eighteen 20° intervals, and then we averaged the corresponding gamma amplitude for each bin. We computed the phase-amplitude modulation index (MI) by the divergence of the empirical phase-amplitude profile from the uniform distribution. The comodulation plot was obtained by calculating the MI between several band-filtered frequency pairs, representing the results in a 2D heat map. For computing mean resultant vector in the polar histogram, the instantaneous phase of the theta-filtered signal has been calculated based on Hilbert’s transform (Hilbert function in MATLAB). The amplitude of filtered LFP in gamma was obtained by Fourier transform (FFT function of MATLAB). We then calculated the distribution of gamma amplitudes associated with a theta phase. The length of the mean resultant vector was obtained by summing up gamma amplitude bins at the preferred theta phase.

### Statistical analysis

Statistical analysis was performed by GraphPad Prism software (GraphPad Software, USA). The normality of the data distribution within each parameter was verified by Kolmogorov-Smirnov Test. The achieved results were compared by t-test. Data are shown as mean ± SEM. In all experiments, statistical significance was considered as the P < 0.05.

## Results

### Behavioral evaluations

**Fig 2:**
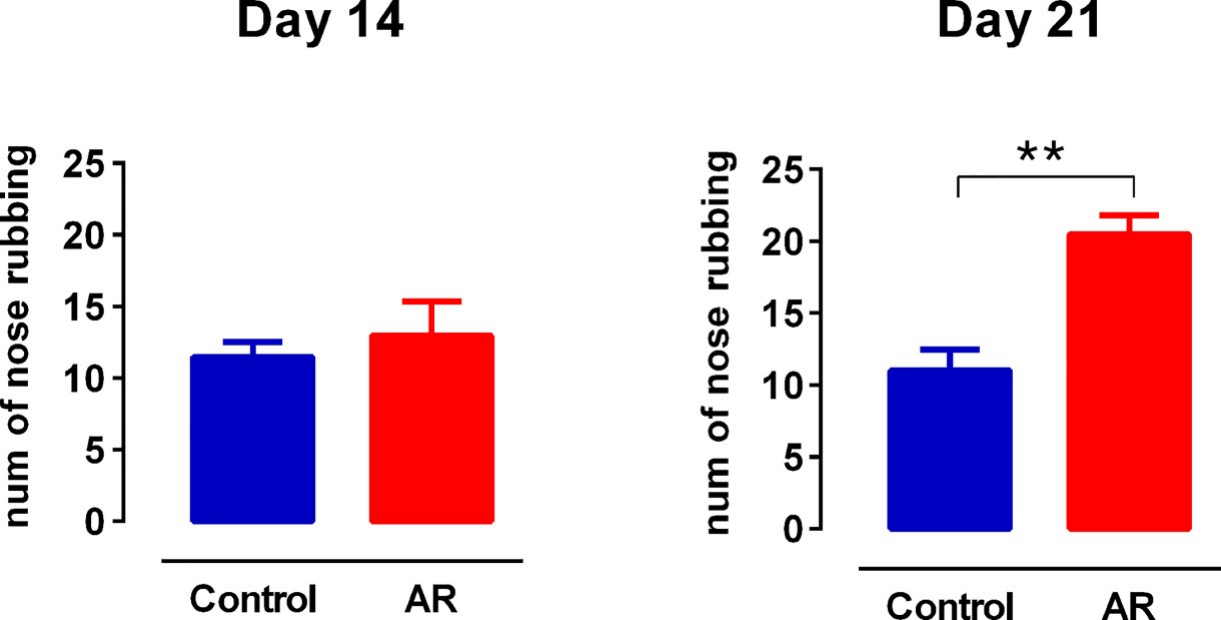
The number of nose rubbing of control and AR groups. Values express as the mean ± SEM. There was a significant increase in the nose rubbing in AR animals compared to the control group on day 21. ** p < 0.01; AR: allergic rhinitis.

**Fig 3:**
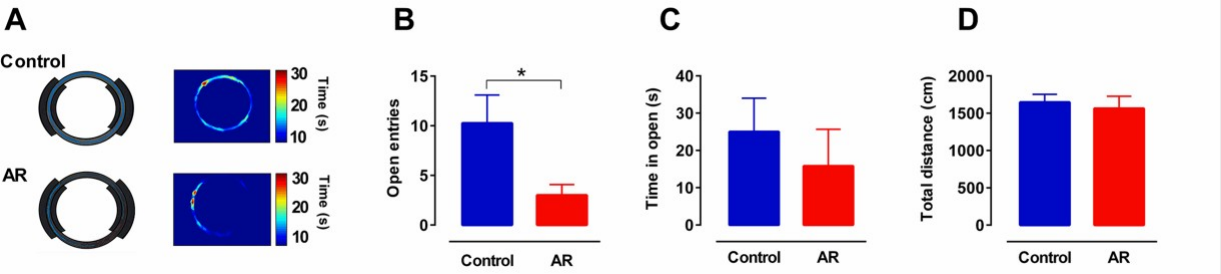
AR induces anxiety-like behavior. (A) Representative tracking areas and heat maps by animals in the EZM for a control (upper panels) and an AR animal (lower panels). Warmer colors represent the animals which spent more time on that sector. (B, C) Spent time and number of entries to the open arena in EZM. (Control: n = 8, OVA: n = 8). (D) Total distance traveled, as an indicator of locomotor activity, in open field test (Control: n = 8, OVA: n = 8). The bar graphs represent mean values ± SEM. Data were analyzed by t-test. * p < 0.05. EZM: elevated zero maze; AR: allergic rhinitis; OB: olfactory bulb; mPFC: medical prefrontal cortex.

The number of nose rubbing motions was assessed in order to confirm the induction of AR model in sensitized rats exposed to OVA. AR model was accomplished on day 21 and the number of nose rubbing motions among AR rats was higher than the control group (p < 0.05), while it was not significant on day 14 (Fig 2). EZM test was performed to quantitate the level of anxiety-like behavior in animals. OVA challenge in sensitized rats significantly reduced the number of open arm entries compared to the control group (p < 0.05) (Fig 3B), indicating enhancement of anxiety-like behavior in AR group. Total distance values in open field test revealed no differences of locomotor activity between groups (Fig 3D).

### Spectral analysis

**Fig 4:**
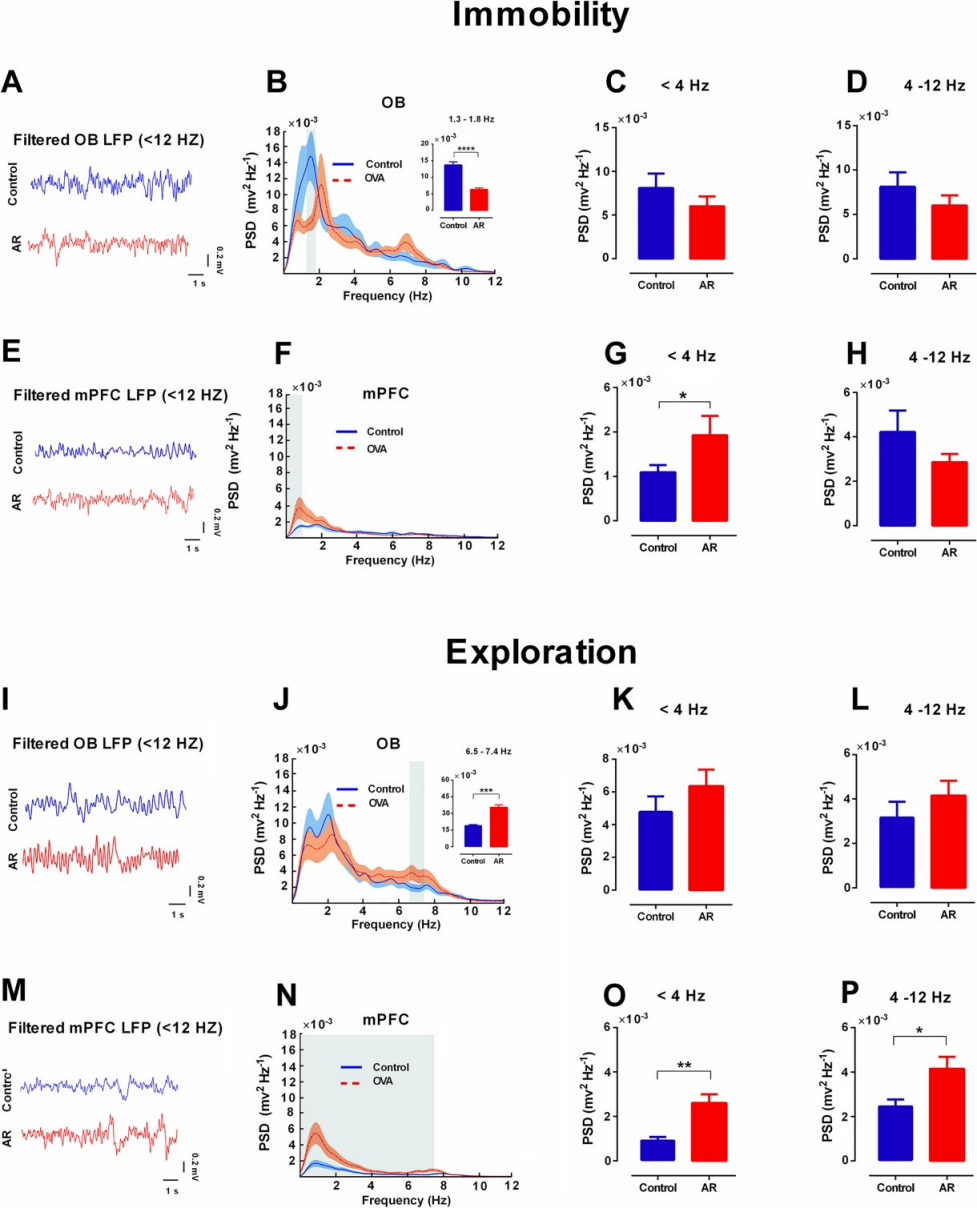
AR increases delta and theta power in OB and mPFC. (A-H) Immobility state. (A, E) Examples of OB and mPFC LFP traces filtered at delta and theta frequencies (< 12 Hz). (B, F) Averaged PSD of recordings in OB and mPFC. Shaded regions denote SEM. (G) AR increases power spectral density of mPFC in delta (< 4 Hz). I-P: Exploration state. (N-P) AR increases delta (< 4 Hz) and theta (4–12 Hz) PSD of mPFC. Inserted panel shows significant differences between AR and control rats in mentioned frequencies. The gray areas indicate significant differences between AR and control rats. Bar graphs represent mean values. Data were analyzed by t-test, n = 8 per group. * p < 0.05, ** p < 0.01 compared to control group. PSD, power spectral density; AR: allergic rhinitis; OB: olfactory bulb; mPFC: medical prefrontal cortex; LFP: local field potential.

Power Spectral analysis (PSD) analysis of LFPs during immobility and exploration showed no significant differences in the mean delta (< 4 Hz) and theta (4–12 Hz) power of OB between both groups (Fig 4C, 4D, 4K and 4L). However, inserted panels (Fig 4B and 4J) exhibited a decrease of power at 1.3–1.8 Hz frequencies (p < 0.0001) and an increase of power at 6.5–7.4 Hz frequencies (p < 0.001) during immobility and exploration, respectively, in the OB of AR animals. LFPs analysis of mPFC revealed that AR animals had more values of power in delta during immobility (p < 0.01), as well as in delta and theta during exploration (p < 0.01 and p < 0.05, respectively) (Fig 4F–4H and 4N–4P).

### Functional connectivity

#### Coherence analysis

**Fig 5:**
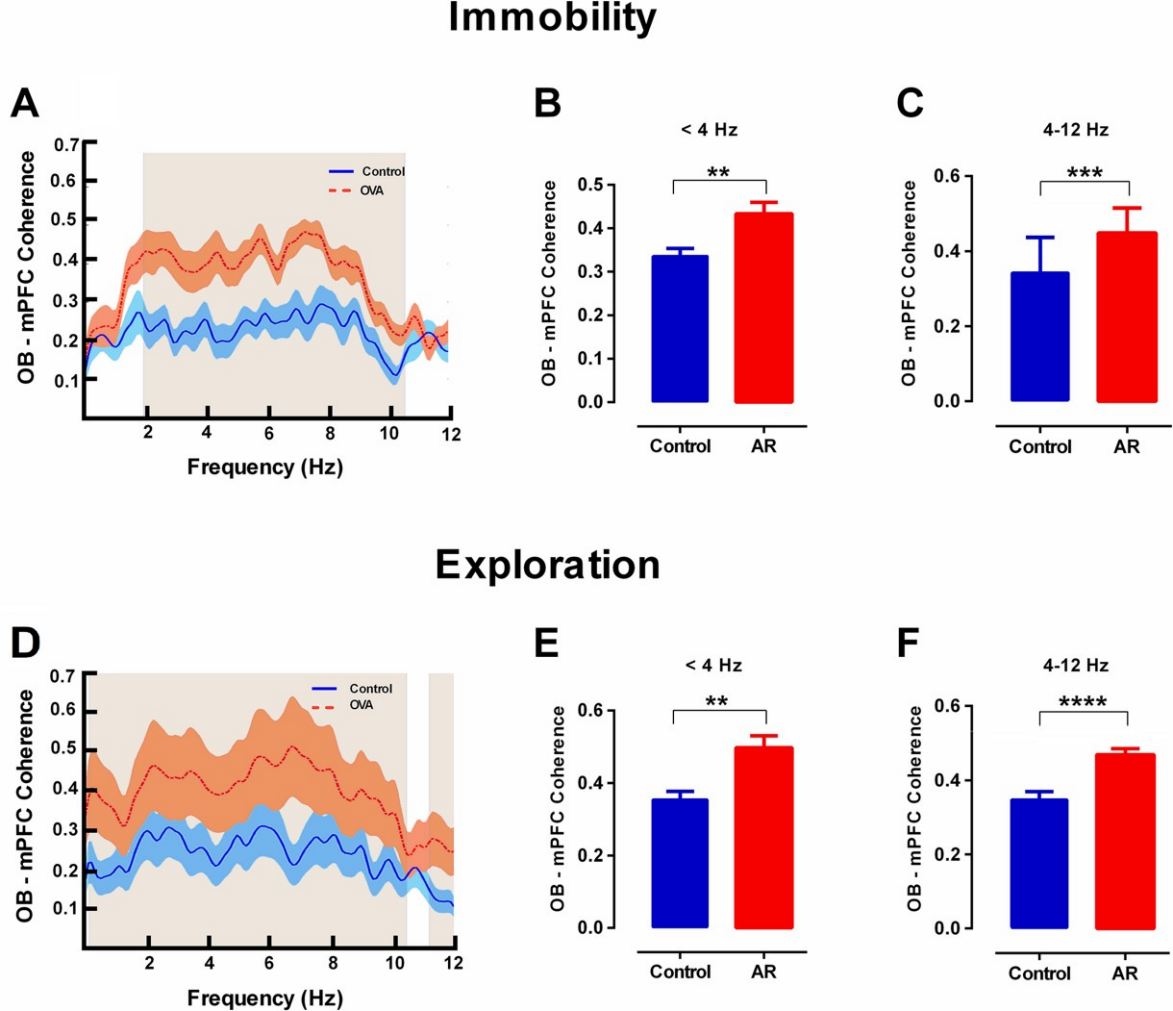
Coherence between OB and mPFC is enhanced at delta and theta frequency in AR rats. (A-C) Immobility state. (A, D) Coherence spectra between OB and mPFC in delta and theta frequencies (< 12 Hz). Shaded area indicates SEM. The gray areas indicate significant differences between AR and control rats. D-F: Exploration state. (B, C, E, F) The bar graphs represent mean values of coherence at delta and theta frequencies. Mean data were analyzed by t-test, n = 8 per group. ** p < 0.01, *** p < 0.05 compared to control group. AR: allergic rhinitis; OB: olfactory bulb; mPFC: medical prefrontal cortex; LFP: local field potential.

In both immobility and exploration states, coherence analysis of the signals, recorded from the OB and mPFC simultaneously, showed higher values for AR rats in delta and theta bands (Fig 5).

#### Cross-correlation analysis

**Fig 6:**
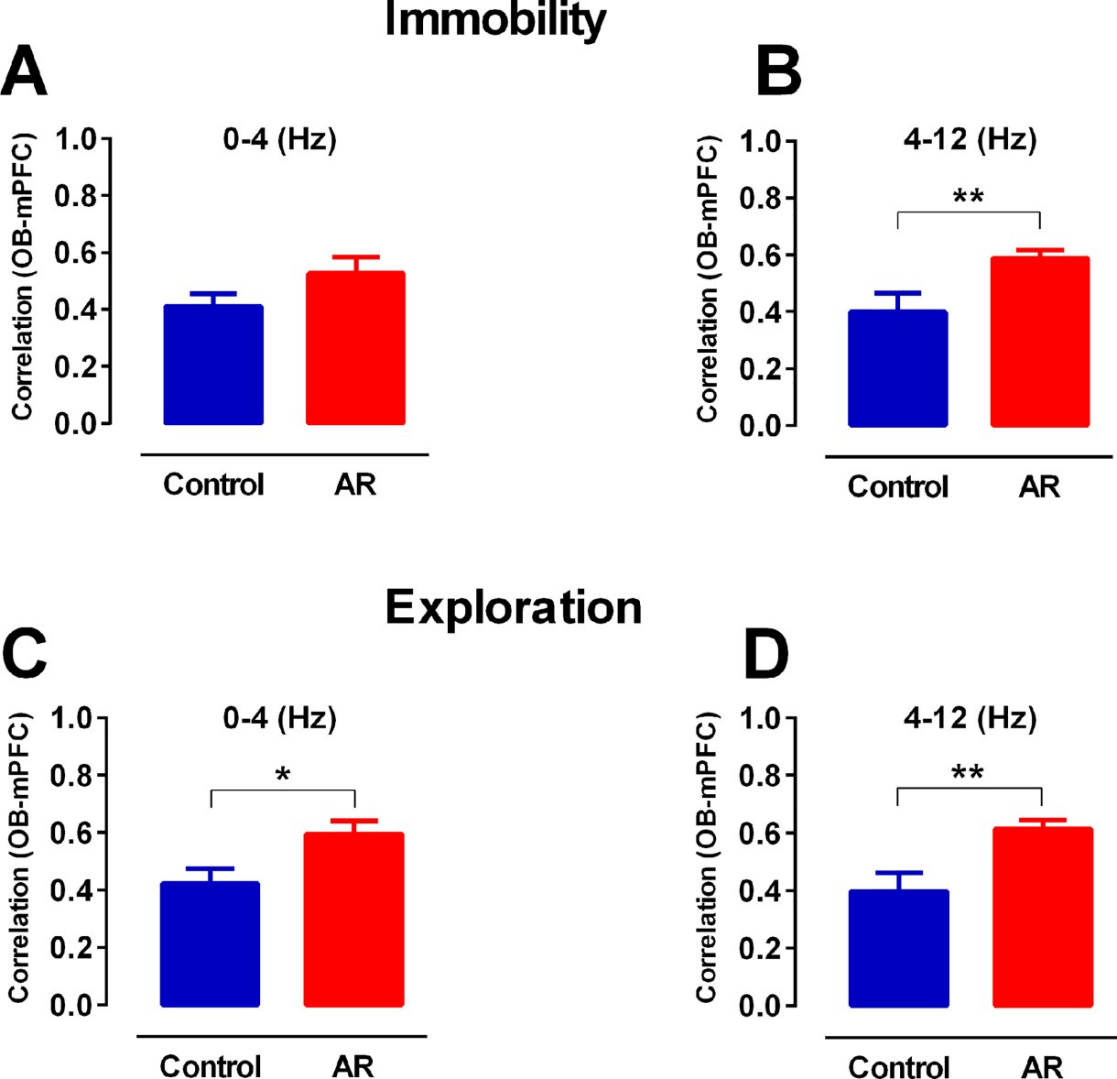
Synchrony of delta and theta activity in OB-mPFC circuit. (A, B) The bar graphs represents cross-correlation coefficients between OB and mPFC in immobility state in delta (< 4 Hz) and theta (4–12 Hz) filtered signals. (C, D) indicates cross-correlation coefficients in delta and theta during exploration state. Data were analyzed by t-test, n = 8 per group. * p < 0.05, ** p < 0.01 compared to control. AR: allergic rhinitis; OB: olfactory bulb; mPFC: medical prefrontal cortex; LFP: local field potential.

We computed cross-correlation to more vastly explore the synchrony of OB and mPFC signals. In AR rats, mean OB-mPFC correlation coefficient for the theta-filtered signals (4–12 Hz) was increased during both immobility and explorations periods (Fig 6B and 6D). We also found a significant cross-correlation increment of delta for exploring AR animals in comparison with control (p < 0.05) (Fig 6C).

#### Theta phase-gamma amplitude coupling

**Fig 7:**
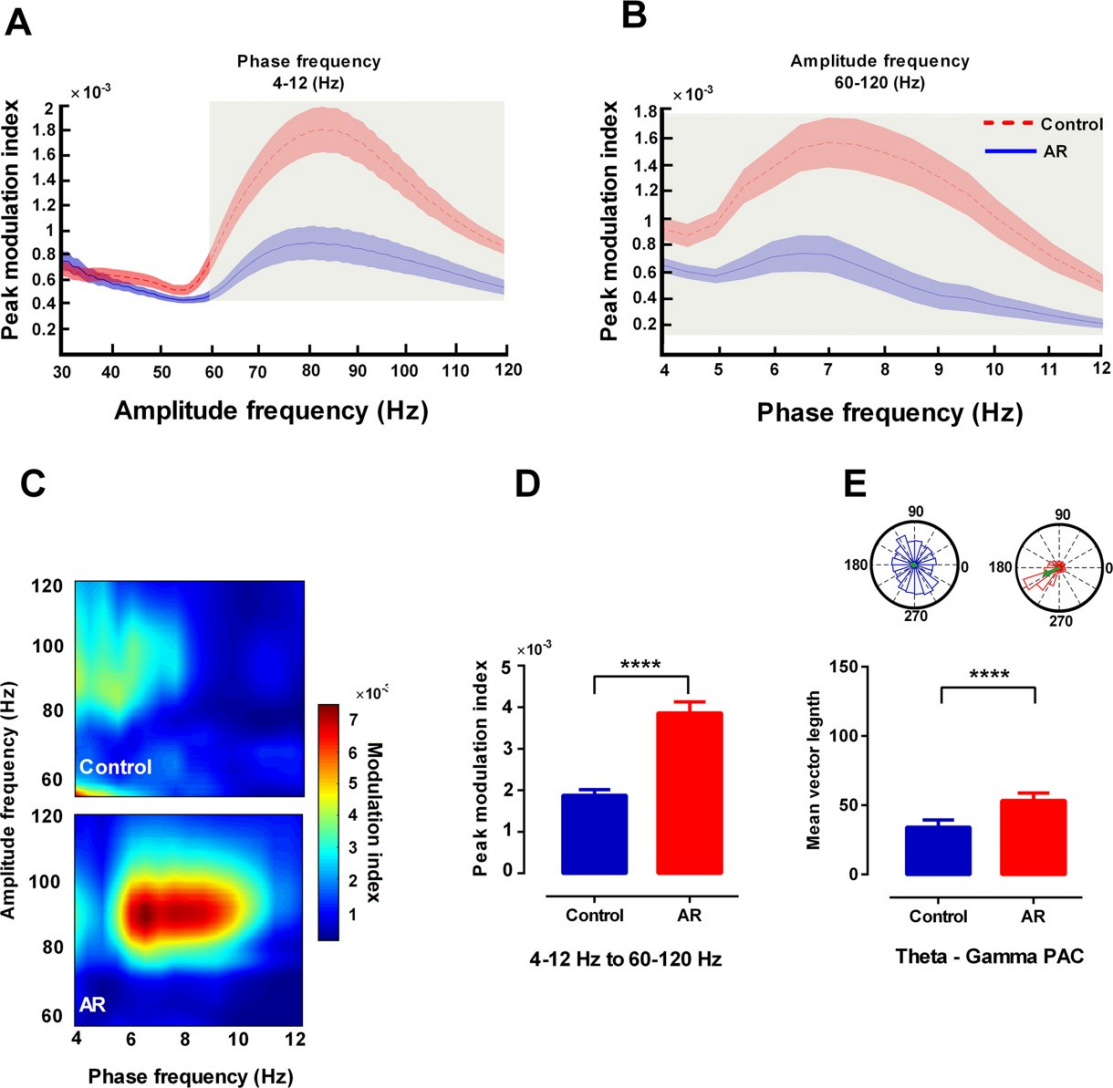
AR increases phase-amplitude coupling of theta and fast gamma oscillations in OB of immobile rats. (A) Peak values of modulation index across amplitude frequencies of theta phase (4–12 Hz) in OB during immobility state. Shaded area indicates standard errors. The gray areas indicate significant differences in fast gamma (60–120 Hz) between AR and control animals. (B) Peak values of modulation index across theta phase frequencies computed for gamma amplitude (60–120 Hz) in OB. (C) The representative comodulogram of modulation index computed for theta phase (4–12 Hz) and gamma (30–120 Hz) in OB. (D) The bar graphs represent mean values of modulation index. (E) The polar plot shows distribution of the gamma (60–120) for the theta cycle phase. Green arrow indicates length of resultant vector. Data were analyzed by t-test, n = 8 per group. **** p < 0.0001 compared to control group. AR: allergic rhinitis; OB: olfactory bulb; LFP: local field potential; PAC: phase amplitude coupling.

Theta oscillations in the brain regions are known to modulate higher local oscillations and are thought to denote a code for neurophysiological processing [32]. Here, we analyzed whether theta could modulate local gamma oscillations in OB and mPFC. We first computed peak values of modulation index across the 30–120 Hz amplitude frequency of theta phase (4–12 Hz), and observed that theta phase of the OB in immobile AR rats modulated fast gamma amplitude (60–120 Hz) significantly higher than that of the control group (p < 0.0001) (Fig 7A and 7B). Hence, for later statistical analysis, peak values of modulation index across theta phase frequencies computed for fast gamma amplitude (60–120 Hz) in OB during immobility state (Fig 7D). Furthermore, the resultant vector declared that theta frequency of OB in the immobile AR animals was strongly coupled to the gamma (60–120 Hz) oscillations (p < 0.0001) (Fig 7E).

**Fig 8:**
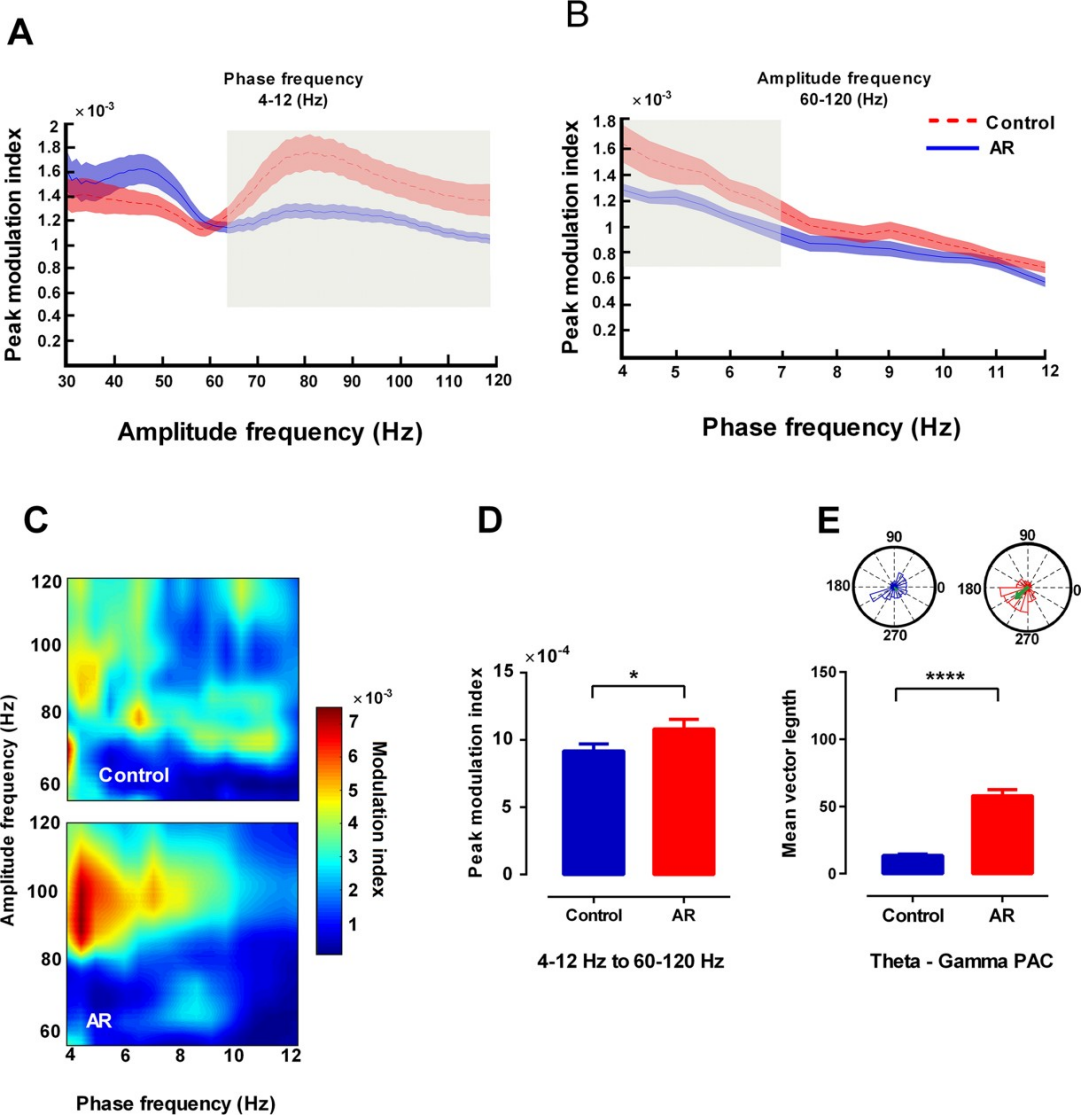
AR increases phase-amplitude coupling of theta and fast gamma oscillations in mPFC of exploring rats. (A) Peak values of modulation index across amplitude frequencies of theta phase (4–12 Hz) in mPFC during exploration state. Shaded area indicates standard errors. The gray area indicates significant differences in fast gamma (60–120 Hz) between AR and control animals. (B) Peak values of modulation index across theta phase frequencies computed for gamma amplitude (60–120 Hz) in mPFC. The gray areas indicate significant differences in 4–7 (Hz). (C) The representative comodulogram of modulation index computed for theta phase (4–12 Hz) and gamma (60–120 Hz) in mPFC. (D) The bar graphs represent mean values of modulation index. (E) The polar plot shows distribution of gamma (60–120) for the theta cycle phase. Green arrow indicates length of resultant vector. Data were analyzed by t-test, n = 8 per group. * p < 0.05, **** p < 0.0001 compared to control group. AR: allergic rhinitis; mPFC: medical prefrontal cortex; LFP: local field potential; PAC: phase amplitude coupling.

In addition, we observed significant theta/gamma phase amplitude coupling in the mPFC during exploration state (p < 0.05) (Fig 8). Phase-amplitude coupling analysis of mPFC in exploring animals revealed that phase of theta at 4–7 Hz in AR rats strongly modulated amplitude of fast gamma (60–120 Hz) compared to control group. Moreover, length of resultant vector in the mPFC of AR rats was higher than that of control group (Fig 8E).

## Discussion

To the best of our knowledge, this is the first report of association between OB-mPFC rhythmic oscillations and anxiety-like behavior in an AR model. Here, we found increased anxiety-like behavior in AR animals accompanied by the increment power of delta and theta in mPFC. In addition, AR-induced anxiety was in association with enhancement of OB-mPFC coupling, as assessed by coherence and cross-correlation analyses. Moreover, depends on the state of activity, phase of theta in OB and mPFC could strongly modulate amplitude of fast gamma (60–120 Hz) in AR animals rather than control group.

A meta-analysis study indicated that there were bidirectional relationships between psychosocial factors and allergic disorders [33]. Although the psychological behaviors could deleteriously involve in the progression of allergic disease, allergic condition had a stronger effect on mental health than that of psychosocial factors on allergic disorder [33]. Behavioral alterations such as anxiety induced by OVA have been well described previously in experimental models of AR. Tonelli et al. demonstrated that OVA challenge could induce anxiety-like behavior in sensitized rodents [17]. Parallel to previous evidence, the present study revealed that the exposure to allergen could increase anxiety-like behavior. Altogether, it may be suggested that anxiety is a complication of allergic reactivity in AR. To explain underlying mechanisms for this effect, former studies proposed that AR induces neuroinflammatory accumulation; and impairment in psychological pathways including hypothalamic-pituitary-adrenal axis, the sympatho-adrenomedullary system, peripheral non-adrenergic non-cholinergic nerves, as well as sensory input. These impairments may mediate a close association between psychosocial problems and allergic disorders [34–38]. On the other hand, psychological stress and emotional alterations in AR patients might affect the level of stress responsiveness as a result of exaggerated cortisol responses, over expression of Th2 cytokine and glucocorticoid resistance [36,39], and consequently might change central nerves system function in response to a peripheral immune challenge [17,36,40].

However, most previous studies merely focused on neuroimmune responses of AR to explain behavioral alterations. In the current study LFP analysis was applied to reveal alterations in the pattern of OB and mPFC oscillations in AR animals. We observed that during immobility state, OB of AR animals represented less power of the narrow frequency sub-band (1.3–1.8 Hz) in delta oscillations. In line with our findings, Ozaki et al. showed that AR reduced power spectra value of OB animals in delta band [19]. The possible reason to this was that the infiltration of inflammatory cells in olfactory mucosa resulted in elevation of the apoptosis in olfactory sensory neurons, which were responsible for delta oscillations [41,42]. Inflammatory reactions might also reduce activity of olfactory pathway as the result of increased mucus secretion, vasodilation and edema in the epithelium of nasal cavity [43]. Moreover, the mPFC was proposed as an essential region in governing the processing of anxiety [23,44]. A recent study found more activity of theta in mPFC within presence of anxious stimuli [45]. Other studies reported that hyperexcitability in the mPFC correlated with heightened anxiety-related behavior [46,47]. In this line, we also observed that anxiety induced by AR was in association with enhancement of delta and theta power in mPFC. Taken together, increased activity in mPFC could be considered as a likely code in anxiety related behaviors.

Olfactory sensory neurons not only relay chemical modality, but also mechanical stimulus induced by nasal airflow enhances the activity of these neurons. Notably, retrograde and anterograde tracing experiments confirmed efferent connection arising from the OB to mPFC [22]. Consistent with this anatomical pathway, optogenetic activation of olfactory sensory neurons entrained the mPFC activity [22]. Andrew et al. recently reported that naris occlusion or pharmacological lesion of the olfactory epithelium significantly c-Fos disrupted the oscillatory circuit of OB-mPFC, suggesting that inputs from olfactory contributed to the PFC activity during emotional reactivity [22]. In this line, frequency and time domain analysis of the present study demonstrated enhancement of OB-mPFC coupling in AR animals which were shown more level of anxiety. It seemed that neuromorphological abnormalities due to chronic stress in the context of brain inflammation could be an underlying possible reason for changes in OB-mPFC coupling in the presence of anxiety induced by AR [48].

Moreover, in AR animals, theta strongly coordinates local gamma activity in OB and mPFC which means a strong local theta/gamma coupling. Phase-amplitude coupling referred to a synchronization of slow waves (SW) phase with power of fast frequency (FW) waves [49]. Several studies analyzed the SW–FW coupling in subjects with anxiety-related pathological conditions. Miskovic et al. suggested that the frontal area of individuals with anxiety disorder exhibited high level of SW-FW phase–amplitude coupling in an anxiogenic situation [50]. Another human study also showed that during anxiogenic condition, subjects who had higher scores of anxiety tended to increase SW-FW coupling in cortical regions around the prefrontal cortex [51]. In line with these studies, our findings demonstrated an enhancement of local phase-amplitude coupling in OB and mPFC in rodents with AR-induced anxiety. Although all neurophysiological concepts of this phenomenon remained unexplored, it was mostly acknowledged that slow oscillations had major role in the timing of neuronal activities and might coordinate functions of brain such as emotional behaviors [52].

We have faced some limitations to interpret entire mechanism. First, we did not record the LFP signals at the same time when the animals were traveling through the EZM. In the animal with AR-induced anxiety, simultaneous record during performance of anxiety-like behavior could provide a deeper insight into the changes of brain network in critical time points. Secondly, our study was limited to evaluate single unit recording which could precisely indicate activity of neurons. Undoubtedly, it would be helpful for the future studies that evaluate circuit of OB-mPFC within anxiogenic condition induced by AR in order to clarify underlying mechanisms of this phenomenon.

## Conclusions

AR can attribute to anxiety-like behavior in association with alterations of LFPs in OB-mPFC circuit. Proposing the role of neuronal pathways between OB and mPFC in presence of AR-induced anxiety opened a door for future studies to realize how these connections might coordinate cognitive and emotional behaviors in allergic conditions. The data can also conclude to make more effective clinical decisions and superior approaches for managing the behavioral impairment of AR as well as other respiratory allergic conditions.

## Supporting information

S1 Data Numerical data underlying Figs. Folder of minimal data set contains raw data in mat and xls format.
(RAR)
